# Update on Immunodeficiency-Associated Vaccine-Derived Polioviruses — Worldwide, July 2018–December 2019

**DOI:** 10.15585/mmwr.mm6928a4

**Published:** 2020-07-17

**Authors:** Grace Macklin, Ousmane M. Diop, Asghar Humayun, Shohreh Shahmahmoodi, Zeinab A. El-Sayed, Henda Triki, Gloria Rey, Tigran Avagyan, Varja Grabovac, Jaume Jorba, Noha Farag, Ondrej Mach

**Affiliations:** ^1^World Health Organization, Geneva, Switzerland; ^2^World Health Organization Regional Office, Cairo, Egypt; ^3^Tehran University of Medical Sciences, Tehran, Iran; ^4^Pediatric Allergy and Immunology Unit, Children's Hospital, Ain Shams University, Cairo, Egypt; ^5^Laboratory of Clinical Virology, World Health Organization Reference Laboratory on Poliomyelitis, Institut Pasteur de Tunis, Tunisia; ^6^World Health Organization Regional Office, Washington, DC; ^7^Division of Viral Diseases, National Center for Immunization and Respiratory Diseases, CDC; ^8^Global Immunization Division, Center for Global Health, CDC; ^9^World Health Organization Regional Office, Manila, Philippines.

Since establishment of the Global Polio Eradication Initiative* in 1988, polio cases have declined >99.9% worldwide; extensive use of live, attenuated oral poliovirus vaccine (OPV) in routine childhood immunization programs and mass campaigns has led to eradication of two of the three wild poliovirus (WPV) serotypes (types 2 and 3) (*1*). Despite its safety record, OPV can lead to rare emergence of vaccine-derived polioviruses (VDPVs) when there is prolonged circulation or replication of the vaccine virus. In areas with inadequate OPV coverage, circulating VDPVs (cVDPVs) that have reverted to neurovirulence can cause outbreaks of paralytic polio (*2*). Immunodeficiency-associated VDPVs (iVDPVs) are isolated from persons with primary immunodeficiency (PID). Infection with iVDPV can progress to paralysis or death of patients with PID, and excretion risks seeding cVDPV outbreaks; both risks might be reduced through antiviral treatment, which is currently under development. This report updates previous reports and includes details of iVDPV cases detected during July 2018–December 2019 (*3*). During this time, 16 new iVDPV cases were reported from five countries (Argentina, Egypt, Iran, Philippines, and Tunisia). Alongside acute flaccid paralysis (AFP) surveillance (*4*), surveillance for poliovirus infections among patients with PID has identified an increased number of persons excreting iVDPVs (*5*). Expansion of PID surveillance will facilitate early detection and follow-up of iVDPV excretion among patients with PID to mitigate the risk for iVDPV spread. This will be critical to help identify all poliovirus excretors and thus achieve and maintain eradication of all polioviruses.

## Classification of VDPVs and Identification of iVDPV

Poliovirus isolates are grouped into three categories: WPV, Sabin-related poliovirus, and VDPV (*3*). Sabin-related viruses have limited divergence in the capsid protein (VP1) nucleotide sequences from the corresponding OPV (Sabin) strain: poliovirus types 1 and 3 (PV1 and PV3) are ≤1% divergent; poliovirus type 2 (PV2) is ≤0.6% divergent. VDPVs have clinical characteristics similar to those of WPV. VDPVs are >1% divergent (from PV1 and PV3) or >0.6% divergent (from PV2) in VP1 nucleotide sequences from the corresponding OPV strain (*4*). VDPVs are further classified as 1) circulating vaccine-derived polioviruses (cVDPVs), when there is evidence of community transmission; 2) iVDPVs, when they are isolated from persons with PIDs; and 3) ambiguous VDPVs (aVDPVs), when isolated from persons with no known immunodeficiency and when there is no evidence of community transmission or when isolates from sewage are not genetically linked to other known VDPVs and whose source is unknown (*3*).

A healthy person typically clears poliovirus infection within 6 weeks. However, in persons with PIDs, an inability to mount an adequate humoral immune response can result in persistence of intestinal infection with poliovirus and prolonged viral shedding (*5*,*6*). The iVDPV case definition is a laboratory-confirmed VDPV infection in a person of any age who has a primary humoral (B-cell) or combined humoral and cellular (B- and T-cell) immunodeficiency disorder (*6*). An iVDPV infection is persistent if VDPV is excreted for >6 months and chronic if excreted for >5 years (*6*).

## Summary of iVDPV Epidemiology, 1961–2019

The World Health Organization (WHO) has compiled reports of iVDPV excretion since 1961 (*6*). As of May 2020, a total of 149 iVDPV cases had been reported to WHO from January 1961 through December 2019 (Table 1). These cases were detected through AFP surveillance (when paralysis occurred before PID was diagnosed) and by reports of iVDPV isolation from fecal specimens (when stool cultures were obtained from patients with suspected or diagnosed PID to detect enterovirus infection). The number of reported cases has increased over time: 66% of cases were detected during 2010–2019. Most onsets occurred in children aged <2 years (59%); 60% of cases were in males; and 64% of patients had acute flaccid paralysis (AFP) as the first sign. The most common PID diagnoses were various antibody disorders, severe combined immunodeficiency disorder (SCID), and common variable immunodeficiency disorder.

**TABLE 1:** Summary of 149 immunodeficiency-associated vaccine-derived poliovirus (iVDPV) cases reported in the World Health Organization (WHO) iVDPV registry — worldwide, January 1, 1961–December 31, 2019*

Characteristic	No. (%)
iVDPV cases reported to WHO (1961–2019)	149 (100)
**Period detected**	
1961–2000	19 (12.8)
2001–2010	31 (20.8)
2011–2020	99 (66.4)
**WHO region**	
African	10 (6.7)
Eastern Mediterranean	74 (49.7)
European	16 (10.7)
Americas	18 (12.1)
South-East Asian	15 (10.1)
Western Pacific	16 (10.7)
**Sex**	
Female	64 (40.6)
Male	85 (59.4)
**Acute flaccid paralysis**	
Yes	95 (63.8)
No	51 (34.2)
Unknown	3 (2.0)
**Age group at onset (yrs)**	
<1	86 (59.3)
1–5	40 (27.6)
>5	19 (13.1)
**Immunodeficiency category**	
Antibody disorders (HGG, AGG, XLA)	39 (28.1)
Common variable immunodeficiency	22 (15.8)
SCID and other combined humoral/T-cell deficiencies	46 (33.1)
Other (MHC class II deficiency, centromere instability, ICF syndrome)	20 (14.4)
Unknown	12 (8.6)
**Serotype**	
1	27 (18.1)
2	83 (55.7)
3	33 (22.1)
1 and 2	3 (2.0)
2 and 3	3 (2.0)
**Outcome**	
Alive	16 (10.7)
Alive and stopped excreting	52 (34.9)
Dead	65 (43.6)
Unknown/Lost to follow-up	16 (10.7)

**Abbreviations:** AGG = agammaglobulinemia; HGG = hypogammaglobulinemia; ICF = centromeric region instability, facial anomalies syndrome; MHC = major histocompatibility complex; SCID = severe combined immunodeficiency; XLA = X-linked agammaglobulinemia.

*Data as of May 17, 2020.

During the reporting period, iVDPV type 2 (iVDPV2) has been the most prevalent serotype (56%), followed by iVDPV type 3 (iVDPV3) (23%) and iVDPV type 1 (iVDPV1) (17%), with 4% heterotypic mixtures (types 1 and 2 in 2% of cases and types 2 and 3 in 2%). Because WPV type 2 had been eradicated, in April 2016, all 155 OPV-using countries and territories switched from trivalent OPV (tOPV, containing types 1, 2, and 3 Sabin strains) to bivalent OPV (bOPV, containing types 1 and 3 Sabin strains), to reduce the risk for paralytic disease from type 2 OPV (from vaccine-associated paralytic polio, which rarely occurs in OPV recipients and their susceptible close contacts; and from VDPV) (*7*). Since the tOPV-to-bOPV switch, the incidence of iVDPV2 cases has declined substantially, with iVDPV1 and iVDPV3 now the most prevalent serotypes (Figure). During 2000–2016, an average of 7.7 cases of iVDPV2 were identified per year (total = 54), compared with 0.67 cases per year (two cases) during 2017–2019. At the most recent follow-up, 16 patients (11%) were alive and still excreting iVDPV, 52 (35%) were alive and had stopped excreting, 65 (44%) had died, and 16 (11%) were lost to follow up (Table 1).

**FIGURE:**
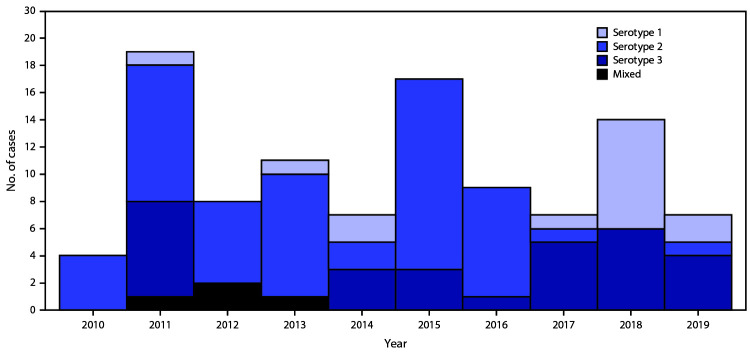
Immunodeficiency-associated vaccine-derived poliovirus (iVDPV) cases reported, by year and VDPV serotype — worldwide, 2010–2019

## Reported iVDPV Cases, July 1, 2018–December 31, 2019

During July 2018–December 2019, 16 new iVDPV cases were reported from five countries (Argentina, Egypt, Iran, Philippines, and Tunisia) (Table 2). These cases included eight iVDPV1 cases, seven iVDPV3 cases, and one iVDPV2 case, with no heterotypic mixtures. The cases are described below.

**TABLE 2:** Immunodeficiency-associated vaccine-derived polioviruses (iVDPVs) detected — worldwide, July 2018–January 2020*

Country	Year detected	Source	PID diagnosis	First positive patient isolate	Serotype	Caspid protein VP1 divergence from Sabin OPV strain^†^ (%)	National 3-dose OPV coverage (%)^§^	Estimated VDPV replication duration^¶ ^(months)	Outcome**
Argentina	2018	AFP case	AGG	Nov 20, 2018	3	1.3	84	21	Stopped excreting
Egypt	2018	Non-AFP case	CID	Jul 15, 2018	3	1.6	95	10	Stopped excreting
Egypt	2018	Non-AFP case	MHC II deficiency	Aug 23, 2018	1	1.7	95	17	Died
Egypt	2018	Non-AFP case	CID	Sep 13, 2018	1	3.6	95	12	Stopped excreting
Egypt	2018	AFP case	SCID	Oct 18, 2018	1	2.6	95	4	Died
Egypt	2018	Non-AFP case	MHC II deficiency	Dec 16, 2018	3	1.6	95	4	Died
Egypt	2018	Non-AFP case	SCID	Dec 25, 2018	1	1.4	95	22	Alive and excreting
Iran	2018	Non-AFP case	SCID	Aug 14, 2018	1	1.0	99	6	Died
Iran	2018	AFP case	B-cell deficiency	Nov 23, 2018	1	1.6	99	22	Alive and excreting
Egypt	2019	Non-AFP case	Unknown	Feb 03, 2019	3	1.4	95	4	Stopped excreting
Egypt	2019	Non-AFP case	SCID	Mar 13, 2019	1	3	95	13	Alive and excreting
Egypt	2019	Non-AFP case	SCID	Jun 18, 2019	3	2.0	95	12	Alive and excreting
Egypt	2019	AFP case	SCID	Aug 28, 2019	3	1.9	95	6	Stopped excreting
Iran	2019	AFP case	AGG	Jul 11, 2019	1	1.3	99	10	Alive and excreting
Philippines	2019	AFP case	Hypokalemia and infectious diarrhea	Aug 29, 2019	2	7.6	66	60	Alive and excreting
Tunisia	2019	AFP case	MHC II deficiency	Mar 12, 2019	3	4.1	97	18	Stopped excreting

**Abbreviations:** AFP = acute flaccid paralysis; AGG = agammaglobulinemia; CID = combined immunodeficiency disorder; MHC = major histocompatibility complex; OPV = oral poliovirus vaccine; PID = primary immunodeficiency disorder; SCID = severe combined immunodeficiency disorder.

* Data as of May 17, 2020.

^†^ Percentage of divergence is estimated from the number of nucleotide differences in the capsid protein VP1 region from the corresponding parental OPV strain in the latest iVDPV sequence available.

^§^ Coverage with 3 doses of OPV, based on 2018 data from the World Health Organization (WHO) Vaccine Preventable Diseases Monitoring System (2018 global summary) and WHO-United Nations Children’s Fund coverage estimates https://www.who.int/gho/immunization/poliomyelitis/en/. National data might not reflect weaknesses at subnational levels.

^¶^ Duration of iVDPV replication was estimated from clinical record by assuming that exposure was from last known receipt of OPV (or date of birth where vaccination data was not available).

** Outcome as of last reported information.

**Argentina (one case)**. In 2018 AFP occurred in a girl aged 9 months who had previously received 2 inactivated poliovirus vaccine doses and 1 bOPV dose in November 2017. In November 2018, iVDPV3 (1.4% VP1 divergence) was detected in a stool specimen. The most recent detection (2.9% VP1 divergence) was collected in August 2019; specimens collected since have been negative, the latest in November 2019. This patient had a diagnosis of agammaglobulinemia.

**Egypt (10 cases).** During July–December 2018, the PID surveillance project in Egypt identified six iVDPV infections, one in a patient who had developed AFP; two cases were iVDPV3 and four iVDPV1. Follow-up revealed that three patients had died, two patients stopped shedding, and one patient shedding iVDPV1 with 2.6% VP1 divergence continued to shed the virus for 22 months after the last reported bOPV dose. During 2019, four patients with iVDPV infection without AFP were detected; three patients had positive test results for iVDPV3, and one patient had a positive test result for iVDPV1. Two patients with iVDPV3 infection stopped excreting after 4 and 6 months.

**Iran (three cases).** In 2018, three iVDPV1 cases were reported, including a case detected before July 2018 and previously reported. These included cases in a boy aged 8 months with SCID who subsequently died and another in a boy aged 11 months who developed AFP in November 2018 and is continuing to excrete, most recently in April 2020. In July 2019, an iVDPV1 case was reported in a girl aged 7 months who had developed AFP; all seven specimens obtained from this patient contained iVDPV1.

**Philippines (one case)**. An iVDPV2 case was detected in August 2019 in a boy aged 5 years who had received 3 doses of tOPV from 2014 to 2015. At his initial evaluation, he had severe malnutrition, significantly reduced antibody levels, and multiple signs and symptoms pointing to a complex immune disorder; however, no specific PID diagnosis was reported. Follow-up stool specimens collected from September 2019 to May 2020 were positive for VDPV2. Concurrent with the detection of the iVDPV2, a cVDPV2 outbreak was detected in the Philippines (*2*). Current genetic evidence indicates that the virus in the patient with iVDPV2 and cases in the cVDPV2 outbreak have similar genetic distance from parental OPV2 strain (7% VP1 divergence) and might share a common origin.

**Tunisia (one case)**. A boy aged 9 months with human leukocyte antigen (HLA)-class II deficiency developed AFP in March 2019. The infant had previously received inactivated poliovirus vaccine and had no history of OPV vaccination. VDPV3 with 1.3%–4.1% VP1 divergence was detected in stool specimens collected during March–December 2019. The child had stopped excreting by March 2020.

## Discussion

Most countries with AFP surveillance detect iVDPV in paralyzed children who then receive a diagnosis of one of the PIDs. However, many iVDPV cases occur in patients with PID without paralysis, and at present, are only detected through special studies or pilot projects of iVDPV surveillance in children with a diagnosed PID. The increase in the number of reported infections during 2010–2019 is likely a consequence of increased efforts to identify infection among patients with PID and improved methods to detect polioviruses. One half of the detected cases were from the WHO Eastern Mediterranean Region, likely related to more recent focus on PID surveillance in that region as well as higher rates of consanguineous marriages, which lead to higher prevalence rates of PID (*8*). WHO has supported several countries in implementing pilot projects for iVDPV surveillance in children with PID, including Egypt, Iran, Pakistan, Sri Lanka, and Tunisia, and more recently, China and India. Additional countries are being identified in other WHO regions and encouraged to implement systematic surveillance in children with PID and without paralysis. WHO and partners have developed guidelines for iVDPV surveillance in patients with PID that should become an integral part of global poliovirus surveillance (*9*).

Detection of cVDPV2 in the Philippines was associated with detection of VDPV2 infection in an immunodeficient patient. This is the first time that an iVDPV and cVDPV linkage has been described in a large outbreak, and further genetic analysis is in progress. It is, however, unclear how or whether the immunodeficient patient contributed to the cVDPV outbreak. The first identified poliovirus of the cVDPV2 outbreak was detected through environmental surveillance with 7% VP1 divergence from parental Sabin type 2 OPV and multiple amino acid changes. The cVDPV2 outbreak was confirmed by isolation of genetically linked virus from multiple additional sewage samples and AFP cases. A higher proportion of nucleotide substitutions leading to amino acid changes is usually found in genomic sequences of identified iVDPV2 from patients with PID.

Continued progress in the development of antiviral medications effective against polioviruses is needed to eliminate virus shedding in persons identified with persistent and chronic iVDPV infections. Pocapavir (a capsid inhibitor) has been administered on compassionate use basis for several patients excreting iVDPV, with mixed results (*10*). Complete clearing of virus has been observed in some recipients; however, rapid development of poliovirus resistance to Pocapavir has been frequently observed (*10*). Therefore, development of a treatment combining Pocapavir with a protease inhibitor currently called V-7404 that is expected to avoid antiviral resistance is continuing. Intravenous immunoglobulin is available to treat patients with PID and poliovirus (as well as nonpolio enterovirus) infection. While antiviral development continues, intravenous immunoglobulin might improve clinical care. Expansion of PID surveillance will facilitate early detection and follow-up of iVDPV excretion among patients with PID to mitigate the risk for iVDPV spread. This will be critical to help identify all poliovirus excretors and thus achieve and maintain eradication of all polioviruses.

**Summary**

**What is already known about this topic?**

Immunodeficiency-associated vaccine-derived polioviruses (iVDPVs) emerge among persons with primary immunodeficiencies (PIDs) and rarely can persist. Persistent iVDPV infection can result in paralysis and potentially seed community transmission.

**What is added by this report?**

After the 2016 global removal of oral poliovirus vaccine type 2 from routine immunization, the reported incidence of iVDPV type 2 infections markedly declined. Increasing surveillance among patients with PID has identified more iVDPV infections without paralysis.

**What are the implications for public health practice?**

Surveillance for iVDPV infections among patients with PID needs to be strengthened, and development of poliovirus antivirals needs to be accelerated to treat iVDPV infections to achieve and maintain eradication of all polioviruses.

## Footnotes

* http://polioeradication.org.
